# Supplementary figures and images for: Cumulative ROC curves for discriminating three or more ordinal outcomes with cutpoints on a shared continuous measurement scale
Source: PLoS One. 2019 Aug 30;14(8):e0221433. doi: 10.1371/journal.pone.0221433 (PMC6716631; doi:10.1371/journal.pone.0221433)

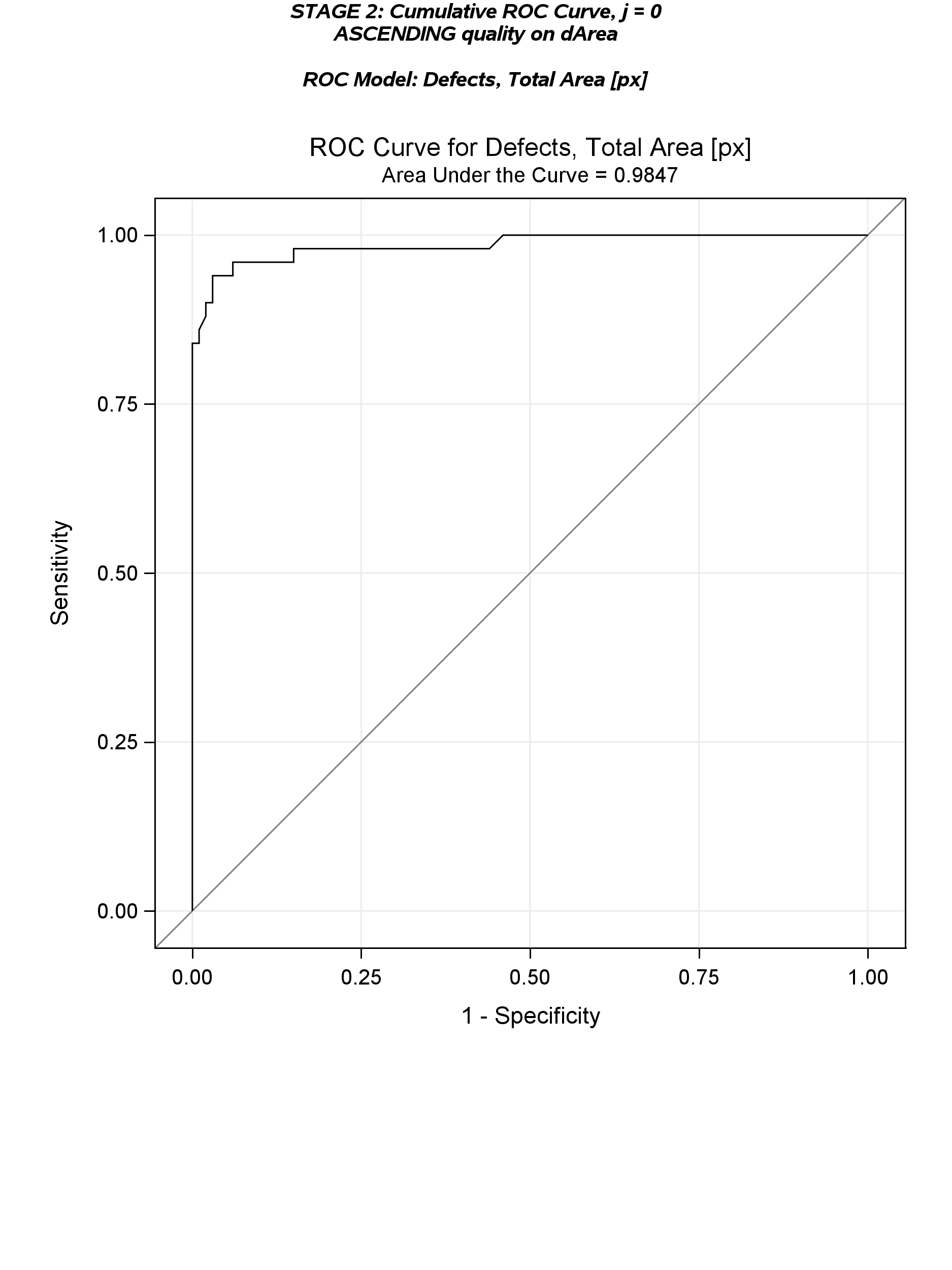

Supplement: S1 Programs — A SAS macro that implements cumulative ROC curve analysis for three-level (ternary) ordinal outcomes, as described in this article. Requires SAS v9.4 or later. (ZIP) [file pone.0221433.s001.zip › cumRoc3/images/cork/ROC0_quality_dArea_PO_2019_DEMO.PNG]

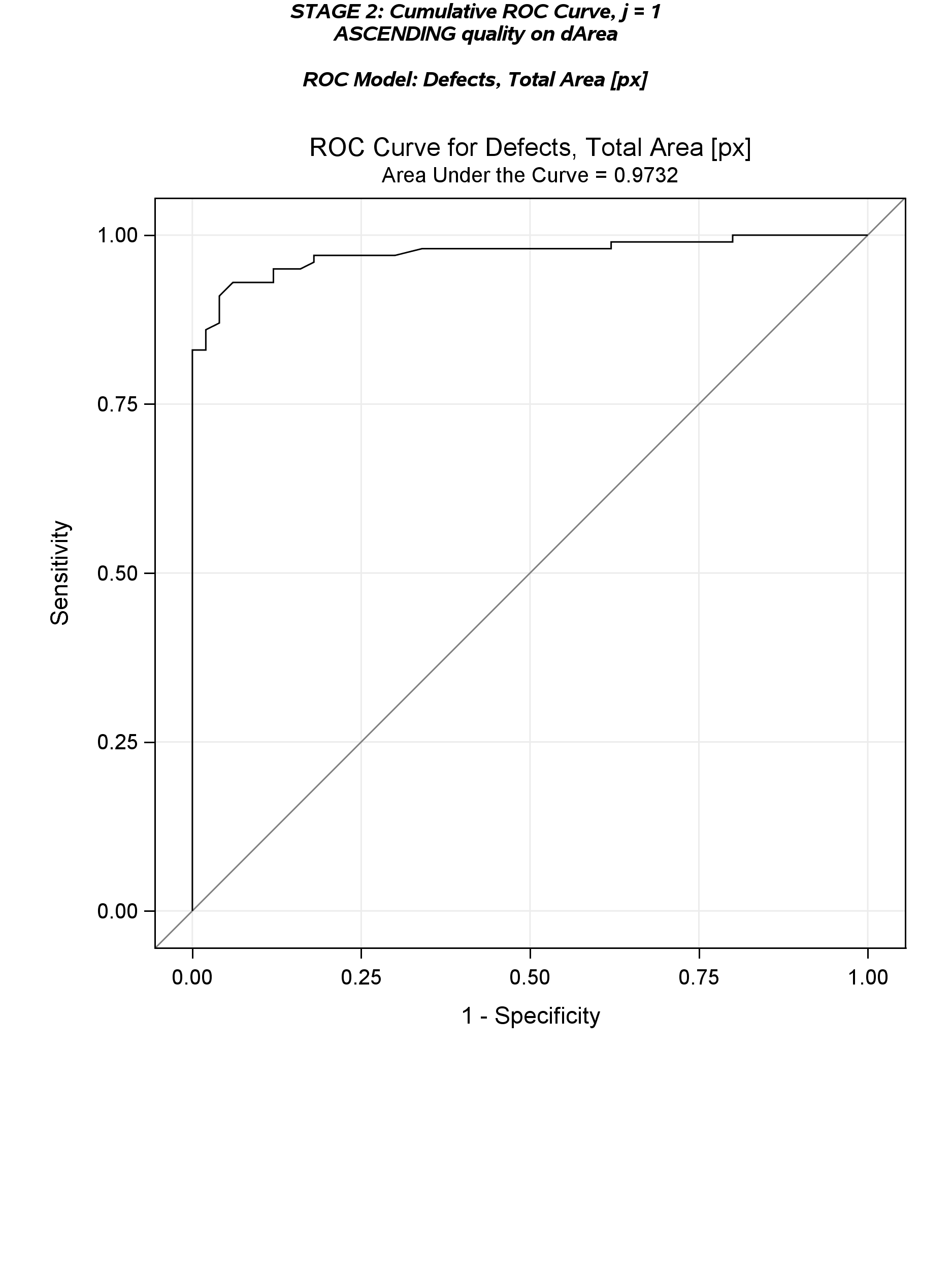

Supplement: S1 Programs — A SAS macro that implements cumulative ROC curve analysis for three-level (ternary) ordinal outcomes, as described in this article. Requires SAS v9.4 or later. (ZIP) [file pone.0221433.s001.zip › cumRoc3/images/cork/ROC1_quality_dArea_PO_2019_DEMO.PNG]

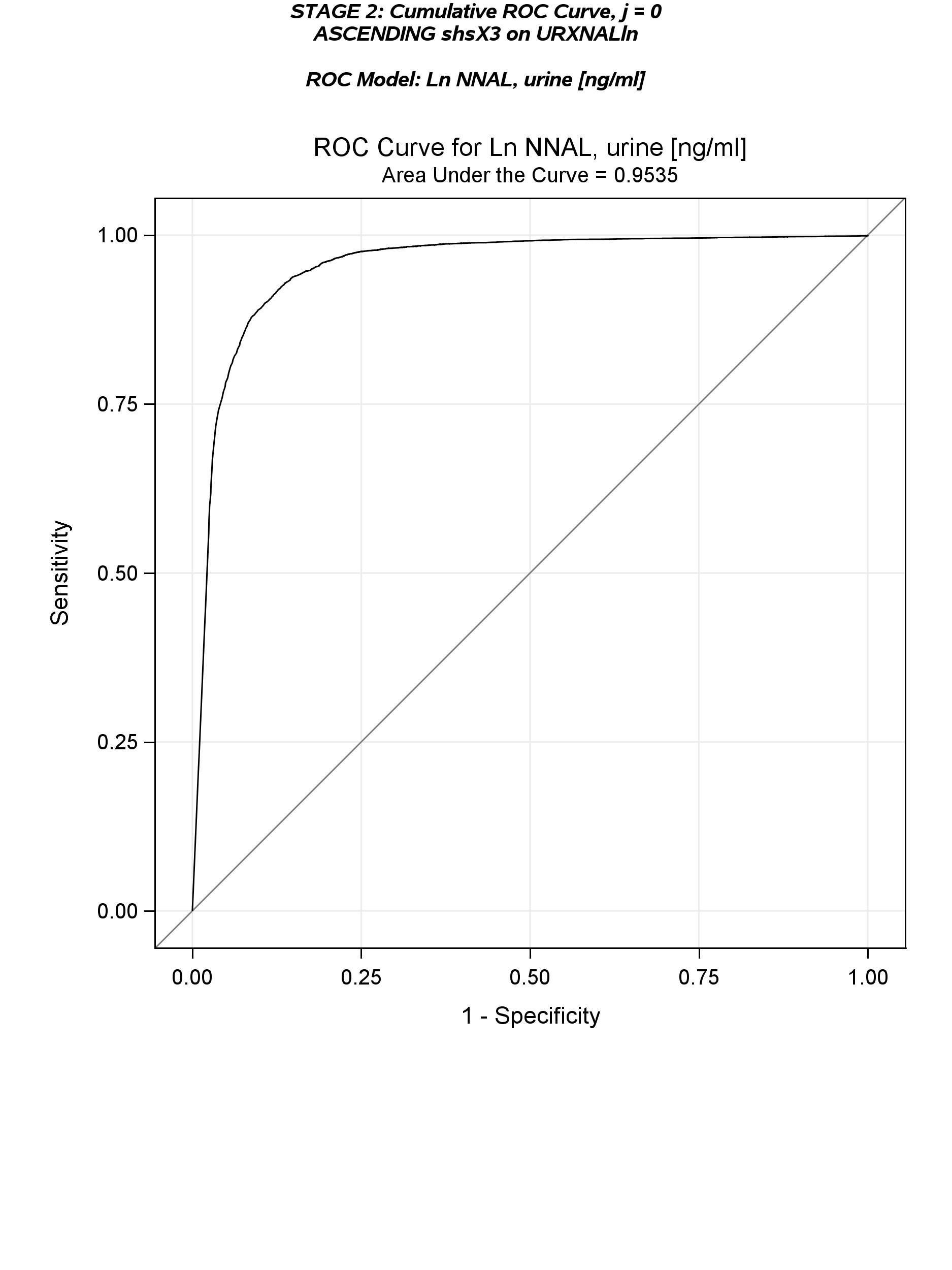

Supplement: S1 Programs — A SAS macro that implements cumulative ROC curve analysis for three-level (ternary) ordinal outcomes, as described in this article. Requires SAS v9.4 or later. (ZIP) [file pone.0221433.s001.zip › cumRoc3/images/nnal/ROC0_shsX3_URXNALln_NPO_2019_DEMO.PNG]

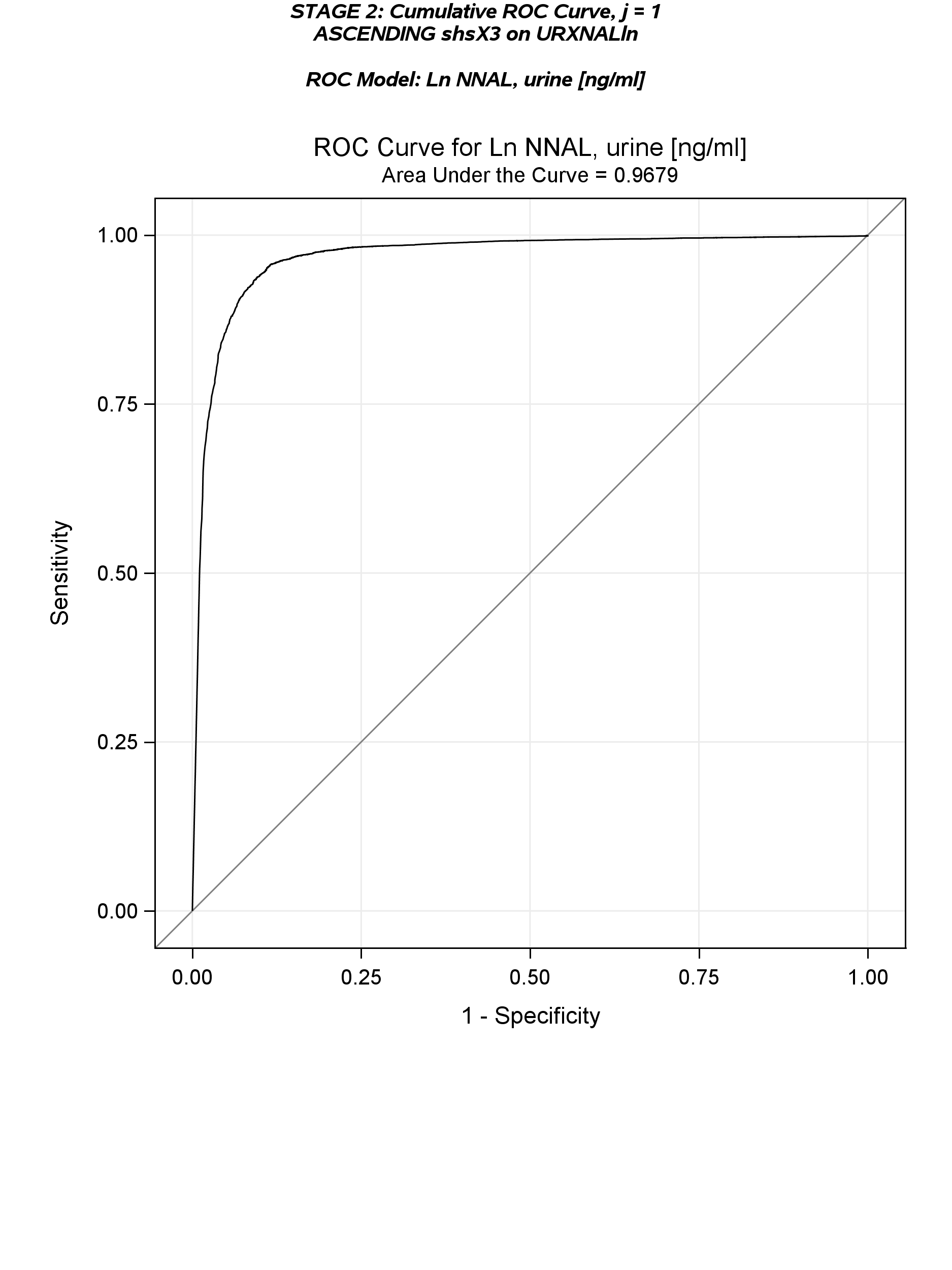

Supplement: S1 Programs — A SAS macro that implements cumulative ROC curve analysis for three-level (ternary) ordinal outcomes, as described in this article. Requires SAS v9.4 or later. (ZIP) [file pone.0221433.s001.zip › cumRoc3/images/nnal/ROC1_shsX3_URXNALln_NPO_2019_DEMO.PNG]
