# Supplementary material for: Cumulative ROC curves for discriminating three or more ordinal outcomes with cutpoints on a shared continuous measurement scale
Source: PLoS One. 2019 Aug 30;14(8):e0221433. doi: 10.1371/journal.pone.0221433 (PMC6716631; doi:10.1371/journal.pone.0221433)
Supplement: Attachment — Submitted filename: PONE-D-19-14500_R1_Response to Reviewers.pdf [file pone.0221433.s004.pdf]

PONE-D-19-14500
"Cumulative ROC curves for discriminating three or more ordinal outcomes with cutpoints on a shared continuous measurement scale"
10 July 2019

10 July 2009
B. Rey deCastro, Sc.D.
Centers for Disease Control and Prevention
Atlanta, Georgia, United States

I am pleased to submit to PLoS ONE my revised manuscript, "Cumulative ROC curves for discriminating three or more ordinal outcomes with cutpoints on a shared continuous measurement scale," for re-consideration as an original Research Article in the area of statistical methods. As requested, I have uploaded both marked-up and unmarked versions of the revised manuscript. The uploaded files are PDFs compiled from a LaTeX source file based on the PLoS ONE LaTex template. The LaTeX source file complies with PLoS ONE editorial specifications and is available upon request.

Today's manuscript has been revised in response to comments received 4 July 2019 from two PLoS ONE reviewers assigned by Academic Editor, Alessandro Parolari, MD, PhD. I am grateful for the reviewers' time in considering this manuscript and their constructive comments, which have substantially improved the manuscript. As requested, I have uploaded a point-by-point response to the reviewers' comments.

In addition to the manuscript, this work includes items uploaded during the original submission: (a) two datasets as ZIP files for inclusion in the Supporting Information; (b) two grayscale figures as TIF files with 300 dpi resolution, 2250 pixel width, two-pixel white border, and labels corresponding to references in their respective captions (color versions available upon request). Forthcoming items are: (c) SAS computer programs implementing the described method, to be available in the Supporting Information and on GitHub and released under a suitable open source license.

Best regards,

B. Rey deCastro, Sc.D.

Reviewer #1

Reviewer #1 Comments to the Author	Author’s Rebuttal
The paper is well written, even if the subject is rather complex. The methodology is sound and the conclusions are in line with the results obtained. I have a few minor comments and modifications to suggest:	The author is grateful for the reviewer’s constructive comments and the consequent improvements to this manuscript.
1. Line 192: it would be helpful to specify on which base (arbitrary or objective?) the three quality levels for the cork stoppers were established.	Agreed. Added: Fifty cork stoppers were quantified in each of three quality levels ≪subjectively assigned by human experts≫
2. The discussion of the simulations is not deep enough (lines 268-274). For instance, the author should discuss the contrasting results of NPO1 and NPO2 (tables 4), where a substantial under- or over-estimation of the parameters occur.	Agreed.<br>Added: ≪The previously noted divergence of the cumulative logit parameters in the NPO2 simulation condition also suggests that caution may be warranted in some non-proportional odds situations, particularly when AUCs of the cumulative ROC curves are widely separated, as in NPO2. If, however, the primary aim is cutpoint estimation, the NPO2 condition indicates that estimated cutpoints were robust against divergence in the logit parameters. Moreover, qualitative results from the NPO2 condition regarding cutpoint selection criteria and parametric cutpoints were consistent with those from the proportional odds and NPO1 simulations.≫<br>See also original lines 146 – 150, 164 – 167.
3. The Discussion includes some methodological specification that should be moved to a previous section: for example, 321-327 should be moved before the ‘Simulations’ section.	Agreed. Moved original lines 321 – 327 to just before Simulations section.

Reviewer #1 Comments to the Author	Author’s Rebuttal
4. The same for the last two sentences of the Conclusions, which contain methodological specifications.	Agreed.<br>First sentence, deleted.<br>Second sentence, revised: ≪The author's programs are freely available for download without warranty or guarantee, which implement cumulative ROC curve analysis with parametric cutpoints for ternary ordinal outcomes (J = 3) in version 9.4 of the SAS statistical software.≫
1. Is the manuscript technically sound, and do the data support the conclusions?<br>Yes	NA
2. Has the statistical analysis been performed appropriately and rigorously?<br>Yes	NA
3. Have the authors made all data underlying the findings in their manuscript fully available?<br>Yes	NA
4. Is the manuscript presented in an intelligible fashion and written in standard English?<br>Yes	NA

Reviewer #2

Reviewer #2 Comments to the Author	Author’s Rebuttal
I read with interest the manuscript. It's a very statistical summary of the employment of cumulative ROC curves for discriminating ordinal outcomes. I have no major comment	The author is grateful for the reviewer’s time in considering this manuscript.
1. Is the manuscript technically sound, and do the data support the conclusions?<br>Yes	NA
2. Has the statistical analysis been performed appropriately and rigorously?<br>Yes	NA
3. Have the authors made all data underlying the findings in their manuscript fully available?<br>Yes	NA
4. Is the manuscript presented in an intelligible fashion and written in standard English?<br>Yes	NA
